# Effects of Sulfate Limitation on Photosynthesis and Cell Composition of Unicellular Marine Microalgae of Different Phylogenies

**DOI:** 10.1111/ppl.70401

**Published:** 2025-07-17

**Authors:** Miles Minio, Mariano Battistuzzi, Alessandra Norici, Nicoletta La Rocca, Cristina Pagliano, Caterina Gerotto

**Affiliations:** ^1^ Department of Life and Environmental Sciences Università Politecnica Delle Marche Ancona Italy; ^2^ Department of Biology University of Padova Padova Italy; ^3^ Department of Science and Technological Innovation University of Eastern Piedmont Alessandria Italy

**Keywords:** C assimilation, cell composition, marine microalgae, photosynthesis, sulfur

## Abstract

Sulfur (S) is an essential macroelement for photosynthetic organisms and is acquired as sulfate and assimilated as sulfide into cysteine through a highly demanding reductive process. S is a key component of proteins, lipids, and various other cellular metabolites and plays a direct role in photosynthesis, both in the electron transport and in carbon fixation reactions. Despite such central functions, most of our knowledge on S metabolism is focused on plant species, while in microalgae it is still fragmented, particularly concerning their huge phylogenetic diversity. Here, we investigated responses to continuous low sulfate availability in three marine microalgae, two Chlorophytes, *Tetraselmis suecica* and *Dunaliella salina,* and the diatom *Phaeodactylum tricornutum,* by characterizing their growth, photosynthesis, elemental, and macromolecular composition. As a general trend, all the microalgae acclimated to the low sulfate medium prioritized the allocation of available resources to photosynthesis. By modulating their pigment content per cell and the stoichiometry of their photosynthetic apparatus, S‐limited cells kept in vivo photosynthetic activity close to that of control cultures. Conversely, growth and cell composition were modulated in a species‐specific manner. Results are discussed also in an evolutionary perspective, taking into consideration that, throughout Earth's history, sulfate concentration significantly increased from ancient to modern oceans, and such variation was paralleled by changes in the ecological abundances between algal groups, with the red algae lineage of present‐day oceans supplanting the green algae, more abundant in the past.

## 1 Introduction

Sulfur (S) is a key component of proteins, lipids, and several other cellular metabolites, thus being one of the essential macronutrients for photosynthetic organisms (Takahashi et al. 2011; Gläser et al. 2014). S is first acquired by the cell as sulfate, then reduced to sulfide through a highly demanding reductive process. In eukaryotic cells, these reactions take place mostly in the chloroplast and are among the main sinks for reducing power and ATP produced by photosynthetic light reactions (Takahashi et al. 2011; Giordano and Raven 2014). The sulfide is then used to produce cysteine (Cys), thanks to reactions catalyzed by the serine acetyl transferase (SAT), producing O‐acetylserine, and O‐acetylserine(thiol)Lyase (OAS‐TL), combining sulfide with O‐acetylserine into Cys (Wirtz and Hell 2006; Takahashi et al. 2011).

Most of our knowledge on S assimilation comes from plants and, although enzymatic reactions leading to S assimilation into Cys appear to be quite conserved from algae to plants, some differences may occur among phylogenetic groups when considering the evolutionary origins, number, isoform localization, and regulation of some of the enzymes required for S assimilation (Patron et al. 2008; Hermsen et al. 2010; Takahashi et al. 2011; Bromke et al. 2013; Gläser et al. 2014; Prioretti et al. 2016; Kopriva et al. 2024).

Moreover, the cellular C:S ratio is quite variable among different algal species even when grown in similar conditions. As a general trend, Chlorophytes are characterized by a low amount of S per unit of C, opposite to dinoflagellates and diatoms showing higher S cell quota and thus a lower C:S ratio, while other groups like coccolithophorids show intermediate C:S ratios (Ho et al. 2003). These differences fit quite well with past and modern phytoplankton oceanic distributions (Falkowski et al. 2004; Norici et al. 2005). In Archean oceans, sulfate concentrations were less than 200 μM (Habicht et al. 2002), rose to around 10 mM after the great oxygenation event (2.5 Ga), and remained as low throughout the Palaeozoic (Lenton et al. 2018). During this period, cyanobacteria and green algae were the dominant primary producers in the oceans but their abundance then decreased after the “late Palaeozoic phytoplankton blackout” (Riegel 2008) and, with the rise of S concentrations to ranges of 17–27 mM in the early Mesozoic, comparable to those found in today's oceans of 28 mM, diatoms, dinoflagellates and coccolithophorids supplanted the green algae in abundance (Falkowski et al. 2004; Knoll et al. 2007; Kodner et al. 2008; Johnston et al. 2009; Giordano et al. 2018; Kroeck et al. 2022). It has been hypothesized that one of the factors that may have facilitated this transition in species dominance is the rise of oceanic sulfate concentrations, which may have favored algae with higher S requirements (Ratti et al. 2011).

To maintain an adequate cellular S content, not only sufficient environmental S is necessary, but also the metabolic energy needed for its uptake and assimilation into organic molecules is essential. The metabolic cost of S assimilation accounts for 33 ATP equivalents from sulfate to Cys (Giordano and Raven 2014). This energy requirement, together with the need for cells to allocate ATP and reducing power properly among carbon (C), nitrogen (N), and S assimilation to maintain an appropriate elemental stoichiometry, may lead to a scarce allocation of metabolic energy to S assimilation under suboptimal growth conditions (Giordano and Raven 2014). As such, S assimilation and photosynthesis show a quite complex interdependence. Photosynthesis provides the energy supporting S assimilation. At the same time, S plays a central role in photosynthesis, both in the light reactions, for example in the form of Fe‐S clusters embedded in the Cytochrome *b*_*6*_ *f* (cyt*b*_*6*_ *f*) and Photosystem I (PSI) thylakoid complexes, and in C fixation with Ribulose‐1,5‐Bisphosphate Carboxylase/Oxygenase (RuBisCO) being regarded as one of the largest cell reservoirs of S (Ferreira and Teixeira 1992). Moreover, many enzymes of the Calvin‐Benson cycle are redox‐regulated through Cys residues (Michelet et al. 2013; Gurrieri et al. 2021). The link between S availability and photosynthesis is well exemplified in the microalga *Chlamydomonas reinhardtii*, in which S deprivation has been used to induce hydrogen production (Melis et al. 2000; Antal et al. 2016; Kosourov et al. 2021). Indeed, under S deprivation, the Photosystem II (PSII) activity of this alga is gradually decreased, leading to the establishment of anoxic conditions, essential to prevent the hydrogenase inactivation by oxygen. Yet, these studies rely on acute temporary S deprivation, thus overlooking the effects of long‐term low S availability on photosynthesis. Besides studies on *C. reinhardtii* , which is a freshwater species and thus has adapted to an environment nearly devoid of S (Giordano et al. 2005), those studies available on marine microalgal species subjected to reduced S availability are instead mostly focused on effects other than those on photosynthesis itself. They include research on the activity of enzymes involved in S assimilation or on the effects of S availability on cellular elemental and macromolecular composition (Giordano et al. 2000; Ratti et al. 2011; Prioretti and Giordano 2016). Production of the protective molecule dimethyl‐sulfoniopropionate (DSMP; Norici et al. 2005; Ratti et al. 2011; Bochenek et al. 2013) and the contribution of DSMP as an organic S source in the marine S cycle (Fernandez et al. 2022) have also been investigated.

Here, experiments were performed on three marine microalgae, selected based on their phylogeny. Two are marine green microalgae, namely *Tetraselmis suecica* and *Dunaliella salina*. The former is a member of the Chlorodendrophyceae, an early diverging clade of the Chlorophyta (Leliaert et al. 2012; Fang et al. 2017). The latter is a member of the more recent Chlorophyceae (Leliaert et al. 2012; Fang et al. 2017) and has become a model organism for the study of environmental stress responses due to its ability to grow in hypersaline environments, characterized by rapid shifts in salinity and nutrient depletion due to salts precipitation (Giordano et al. 2000; Monte et al. 2020; Polle et al. 2020). The third species is the model diatom *Phaeodactylum tricornutum* , selected as a member of the red lineage (Falciatore et al. 2020).

The microalgae were acclimated to low sulfate availability and characterized for their growth, cell composition, and photosynthesis, to explore whether and how they acclimate their photosynthetic apparatus to cope with S‐limitation.

## 2 Materials and Methods

### 2.1 Species and Growth Conditions

Cultures of *Tetraselmis suecica* (PLY 305), *Dunaliella salina* (CCAP19/25) and *Phaeodactylum tricornutum* (CCAP1052/6) were grown in AMCONA medium (Fanesi et al. 2014) and kept in controlled conditions: 20°C, 24 h continuous light (Giordano et al. 2000; Ratti et al. 2011), with light intensity set at 50 ( *T. suecica* and *P. tricornutum* ) or 100 ( *D. salina* ) μmol photons m^−2^ s^−1^, hereafter referred to as control (CTR) conditions. For S‐limitation, the medium was modified by substituting sulfate salts with chloride salts, and Na_2_SO_4_ was added at the target sulfate concentration. Acclimation to low sulfate was achieved with subsequent dilutions of the cultures, kept in the same light and temperature conditions as the respective CTR, from 25 mM sulfate of the standard AMCONA medium to the final 50 μM sulfate (hereafter, S‐lim). The intermediate dilution steps were optimized for each species as follows: 25 mM to 500 μM, 100 μM and finally 50 μM sulfate for *T. suecica* ; 25 mM to 500 μM and finally 50 μM sulfate for *D. salina* ; 25 mM to 1 mM, 500 μM, 100 μM and finally 50 μM sulfate for *P. tricornutum* . Cells were acclimated for at least four generations (as in Ratti et al. 2011) to each sulfate concentration before the next dilution as well as to the final concentration of 50 μM before starting the experiments to characterize the cells. Only in the case of *D. salina* , because of its well‐known capacity to grow in hypersaline environments, experiments were performed also on cultures grown with an AMCONA medium enriched in NaCl, hereafter referred to as 3×NaCl, as higher saline concentrations are commonly used for this species (Oren 2014; Park et al. 2015). The respective S‐limited culture was obtained in the 3×NaCl AMCONA medium following the method described above for the standard AMCONA medium.

Culture cell density was measured daily with a CASY TT Cell Counter (Innovatis AG). Growth rates were calculated in the exponential growth phase. Different time ranges were used according to the species/condition: 0–3 days for *T. suecica* ; 0–2 days for *D. salina* CTR and 3×NaCl CTR; 1–8 days for *D. salina* S‐lim and 3×NaCl S‐lim; 0–3 days for *P. tricornutum* .

All the following experiments were performed on cells sampled in the mid‐late exponential growth phase of batch cultures at control (CTR, i.e., replete) or limiting sulfate concentration (S‐lim). Sampling occurred at day 3 of the batch cultures growth, except for *D. salina* S‐lim and 3×NaCl S‐lim, for which sampling was delayed 2 and 3 days, respectively, due to the longer lag phase we detected in these cultures. All measurements were carried out in at least three biological replicates, that is three independent flasks.

### 2.2 Elemental Composition of Microalgal Cells

For C and N quantification, pellets deriving from the cultures were washed three times with an ammonium formate solution, isosmotic to the culture, and dried at 80°C. C and N content of 0.8–1.2 mg samples was then determined with a CHN element analyser (ECS 4010, Costech Italy), connected to an ID Micro EA isotope ratio mass spectrometer (Compact Science Systems, Lymedale Business Centre) to determine the stable carbon isotope (δ^13^C) ratio. Isotopic urea was used as a standard for the analysis.

The cellular content of S and phosphorus (P) was measured through a total reflection X‐ray fluorescence (TXRF) spectrometer (S2 PICOFOX, Bruker AXS Microanalysis GmbH). About 0.6–1.5*10^7^ cells for the two green algae and 1.5–3*10^7^ for *P. tricornutum* were washed thrice in an isosmotic ammonium formate solution and resuspended in 1 mL of grade 1 water. A 0.1 g l^−1^ solution of Ga in 5% HNO_3_ (Sigma‐Aldrich) was added as an internal standard for a final concentration of 5 mg l^−1^ of Ga. The suspension was then vortexed and a 10 μL aliquot was deposited on a quartz sample holder, dried on a heating plate, and quantification of elemental abundances was performed via the spectra 6.1 software (Bruker AXS Microanalysis GmbH).

### 2.3 Cell Protein Content

About 2*10^6^ cells for the two green algae and 3*10^6^ for *P. tricornutum* were pelleted, and their protein content was measured following the Lowry method (Peterson 1977; Waterborg and Matthews 1994). The absorbance of each sample was then measured spectrophotometrically (UV‐1900i, SHIMADZU CORP.) at 750 nm, and the protein content of each sample was calculated by interpolating the sample's absorbance with a standard curve constructed with known concentrations of bovine serum albumin.

### 2.4 Fourier‐Transformed Infrared Spectroscopy (FTIR)

About 0.6–1.5*10^7^ cells for the two green algae and 1.5–3*10^7^ for *P. tricornutum* were pelleted, washed twice with isosmotic ammonium formate solution, and resuspended in 110 μL of ammonium formate for Fourier Transform Infrared spectroscopy (FTIR). 50 μL aliquots were deposited onto a silicon disk and dried at 80°C (Domenighini and Giordano 2009). FTIR spectra were then acquired from whole cells with a Tensor 27 FTIR spectrometer (Bruker Optics). Bands associated with cellular macromolecular pools (proteins, carbohydrates, and lipids) were assigned as described previously (Giordano et al. 2001). In diatoms, the relative amount of silicate, a component of their frustules, was likewise evaluated thanks to a specific band assigned to the Si‐O‐Si bond (Palmucci et al. 2011). Then, the relative abundances of proteins, carbohydrates, and lipids were calculated via band integrals of the deconvolved spectra, with the OPUS 6.5 software (Bruker Optics GmbH).

### 2.5 Pigment Quantifications

Culture samples (0.5–4 mL, depending on the species and growth condition) were pelleted, washed with isosmotic ammonium formate, and resuspended in 80% (v/v) aqueous acetone solution for the green algal species or pure ethanol for *P. tricornutum* . The extracts were then stored in the dark at −20°C till the pellet was completely white. Absorbance spectra of the pigment extracts were recorded spectrophotometrically (UV‐1900i, SHIMADZU CORP.). For *T. suecica* and *D. salina* , Chlorophyll (Chl) *a* and Chl *b* were quantified using equations from Porra et al. (1989) and total carotenoids (Car) according to Wellburn (1994). For *P. tricornutum* , Chl *a* and Chl *c* were quantified according to Ritchie (2006) and fucoxanthin was estimated as in Wang et al. (2018), as done previously (Avilan et al. 2021). Finally, pigment concentrations were expressed as pg cell^−1^.

Pigments were also extracted from *T. suecica* and *D. salina* in 90% (v/v) aqueous acetone solution and from *P. tricornutum* in pure methanol and analysed by high‐performance liquid chromatography (HPLC) to evaluate the relative content of carotenoids. Samples were first washed with grade 1 water to remove salts and then resuspended in the solvent. The individual carotenoids of each sample were determined using an HPLC system (1100 series, Agilent) equipped with a reverse‐phase column (5 μm particle size; 25 × 0.4 cm; 250/4 RP 18 Lichrocart). The elution of the pigments was obtained using the mobile phase consisting of solvent A (86.8% acetonitrile 9.6% methanol, 3.6% Tris–HCl 0.1 M, pH 8) and solvent B (80% methanol, 20% hexane) with a gradient from solvent A to solvent B run from 9 to 12.5 min at a flow rate of 2 mL min^−1^ (Farber and Jahns 1998; Fattore et al. 2021). Once the chromatograms and the absorbance spectra were obtained, they were compared with the spectra and elution times present in the literature to identify each pigment (Jeffrey et al. 1997; Roy et al. 2011). Finally, the relative abundance of each carotenoid was calculated by taking into consideration the area of its peak in the chromatogram over the total areas of all identified carotenoid peaks.

### 2.6 In Vivo Chlorophyll Fluorescence and P700 Analyses

In vivo chlorophyll fluorescence (Chl fluo) and P700^+^ absorption signals were monitored with a Dual PAM‐100 fluorometer (Walz). Samples were concentrated to 6*10^6^ cell ml^−1^ for *D. salina* and *T. suecica* and 2*10^7^ cell ml^−1^ for *P. tricornutum* . Before measurements, samples were dark acclimated for 40 min for *D. salina* and *P. tricornutum* , and overnight for *T. suecica* . The samples were analysed with light curve protocols of 20 steps of increasing actinic light, from 6 to about 2000 μmol photons m^−2^ s^−1^, each lasting 1 min. To avoid any interference of sample stirring on the parameters' calculation, the protocol was customized to allow sample mixing for 40 s during every actinic light step, stopping the stirring 10 s before the saturating pulse was applied to calculate the photosynthetic parameters and switching it on again 10 s after the saturating pulse. Photosynthetic parameters were calculated by the Dual‐PAM‐100 software as follows: Fv/Fm as (Fm−Fo)/Fm; Y(II) as (Fm′−F)/Fm′; NPQ as (Fm−Fm′)/Fm′, Y(I) as (Pm′−P700ox)/Pm.

### 2.7 Total Cellular Protein Extracts, SDS‐PAGE and Western Blotting

For extraction of total proteins, algal pellets from about 100 mL of culture were resuspended in 500 μL extraction buffer (50 mM Tris–HCl pH 8.1, 1 mM Na_2_EDTA, and 10 mM MgCl_2_) and mechanically broken with a potter homogenizer in the case of *D. salina* or an N_2_ cell disruption bomb (4639 Cell Disruption Vessel, Parr Instrument Company) for *T. suecica* and *P. tricornutum* . Then, Triton X‐100 and glycerol were added to final concentrations, respectively, of 0.1% (v/v) and 10% (v/v) to the extracts, and vortexed. After 15 min of incubation in ice, the samples were centrifuged at 2500 g for 10 min at 4°C, and the supernatants were collected and stored at −20°C. Extracts were quantified for their Chl content spectrophotometrically as described above for cell pigments, solubilized in Laemmli buffer (Laemmli 1970) and separated through SDS‐PAGE on a 12% (w/v) polyacrylamide gels, which then were either stained with Coomassie brilliant blue R‐250 or transferred onto PVDF or nitrocellulose membranes for subsequent immunoblotting. Proteins were immunodetected through the alkaline phosphatase conjugate method with specific antisera, the home‐made antibodies for RuBisCO large subunit (RBCL), LHCII, D2, and PSAA proteins (for details see Barbato et al. 1995), LHCF1‐11, OAS‐TL A (Heeg et al. 2008) or commercial anti‐PSAD antibody (AS09461, Agrisera). The antibodies used were polyclonal antibodies raised against plant proteins, except for LHCF1‐11, raised against the LHCF isoforms of *P. tricornutum* (Juhas and Büchel 2012).

## 3 Results

### 3.1 Growth of Microalgae in Control and S‐Limiting Conditions

For this study, three unicellular marine microalgal species were analysed: *T. suecica* and *D. salina* , belonging to early diverging or more recent clades of Chlorophyta, respectively, and the pennate diatom model species *P. tricornutum* . As a control condition, they were cultivated in an artificial seawater medium (AMCONA) containing 25 mM sulfate, in line with that of the present oceans (hereafter CTR). A corresponding medium, modified with reduced sulfate content, was used to induce S‐limiting growth conditions. Preliminary rounds of acclimation allowed us to test a range of sulfate concentrations lower than 25 mM of CTR and to select for further experiments a sulfate concentration of 50 μM (hereafter, S‐lim).

**FIGURE 1:**
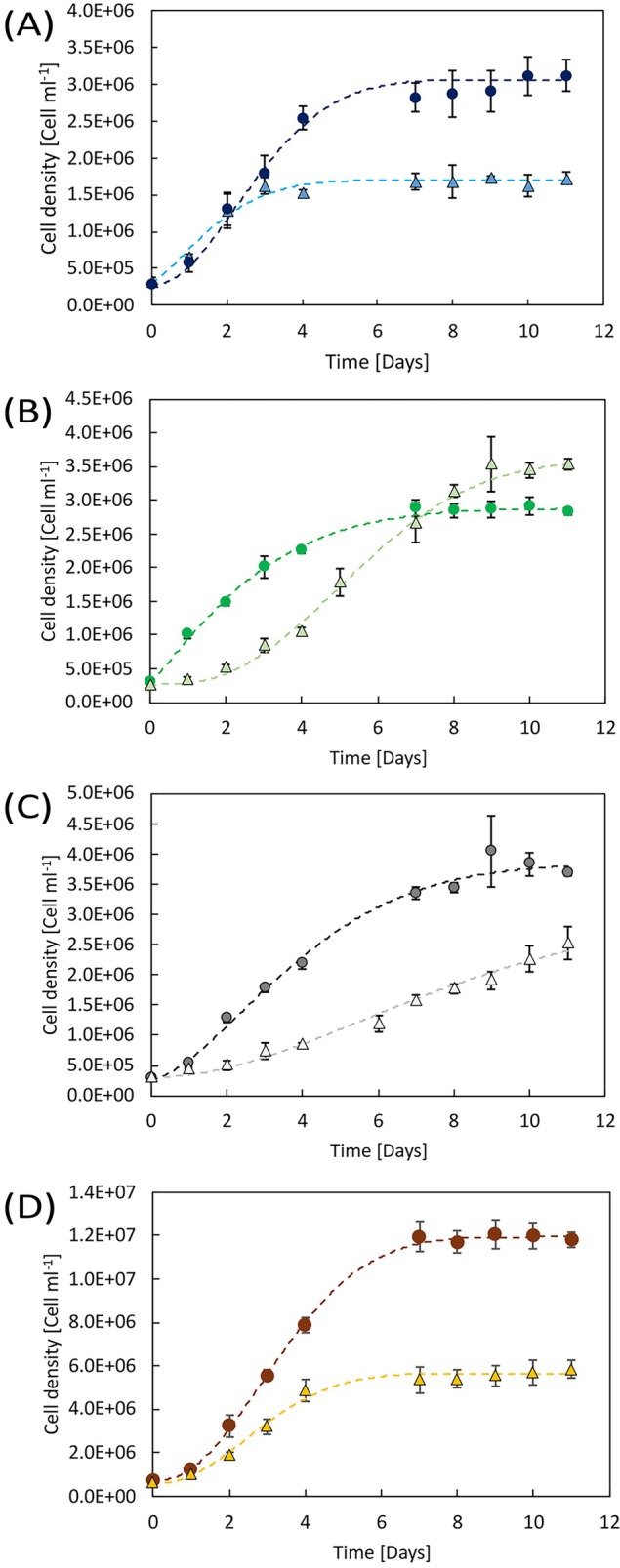
Growth curves of *T. suecica, D. salina* and *P. tricornutum* cells grown in control or S‐limiting conditions. Panels show the growth of *T. suecica* in blue (A), *D. salina* in green (B), *D. salina* 3×NaCl in gray (C), and *P. tricornutum* in orange (D). In all panels, the control (CTR) culture is shown in circles and darker colors, and the S‐limited (S‐lim) culture in triangles with lighter colors. Data is shown as mean ± standard deviation of at least three independent replicates. The corresponding growth rates calculated in the exponential phase and the cell density values in the stationary phase are reported in Table 1.

The growth of batch cultures of *T. suecica* in CTR conditions or after acclimation to 50 μM sulfate (S‐lim) was roughly comparable during the first days of exponential growth, except for a slight decrease in the growth rate for S‐lim cells (Figure 1A, Table 1). However, after day 3, the S‐lim cultures entered the stationary phase, while the CTR cultures continued to grow, reaching higher cell densities (Figure 1A, Table 1).

**TABLE 1:** Impact of S‐limitation on cell growth and composition.

	*T. suecica*		*D. salina*	
	CTR	S‐lim	CTR	S‐lim
Growth rate (day^−1^)	0.64 ± 0.03	0.55 ± 0.06*	0.81 ± 0.05	0.31 ± 0.01***
Max cell density (cells ml^−1^, day 11)	3.12 × 10^6^ ± 2.15 × 10^5^	1.72 × 10^6^ ± 7.98 × 10^4^***	2.82 × 10^6^ ± 4.33 × 10^4^	3.53 × 10^6^ ± 8.24 × 10^4^**
Cell dry weight (pg cell^−1^)	93.4 ± 18.8	82.1 ± 7.1	106.2 ± 17.8	100.4 ± 14.3
Cell vol (fl)	445 ± 35	493 ± 27	415 ± 7	463 ± 21**
%C on DW	49.7 ± 3.6	49.9 ± 1.1	49.1 ± 1.6	47.1 ± 1.3*
%N on DW	4.6 ± 1.1	6.0 ± 0.5***	3.2 ± 0.2	3.4 ± 0.5
%S on DW	0.53 ± 0.12	0.54 ± 0.11	0.21 ± 0.05	0.09 ± 0.02*
%P on DW	0.24 ± 0.05	0.36 ± 0.11*	0.10 ± 0.03	0.06 ± 0.01*
δ^13^C	−15.0 ± 2.4	−25.6 ± 0.7***	−14.4 ± 1.1	−21.6 ± 3.7**

	*D. salina* 3×NaCl		*P. tricornutum*	
	CTR	S‐lim	CTR	S‐lim
Growth rate (day^−1^)	0.77 ± 0.04	0.2 ± 0.01**	0.70 ± 0.02	0.56 ± 0.05*
Max cell density (cells ml^−1^, day 11)	3.68 × 10^6^ ± 5.32 × 10^4^	2.52 × 10^6^ ± 2.75 × 10^5^**	1.18 × 10^7^ ± 3.60 × 10^5^	5.83 × 10^6^ ± 4.09 × 10^5^***
Cell dry weight (pg cell^−1^)	102.4 ± 7.5	135.1 ± 6.4**	10.8 ± 3.2	39.0 ± 7.5**
Cell vol (fl)	621 ± 24	656 ± 13	86 ± 15	113 ± 7*
%C on DW	49.9 ± 1.4	45.5 ± 1.6*	52.3 ± 0.5	50.0 ± 1.4
%N on DW	5.1 ± 0.4	3.6 ± 0.4*	7.9 ± 0.1	7.1 ± 1.0
%S on DW	0.18 ± 0.03	0.06 ± 0.01*	0.43 ± 0.13	0.21 ± 0.05*
%P on DW	0.07 ± 0.02	0.09 ± 0.02	0.31 ± 0.11	0.16 ± 0.04
δ^13^C	−13.9 ± 0.2	−23.5 ± 0.5***	−24.2 ± 1.0	−33.7 ± 1.5***

*Note:* The table reports the growth rate, calculated from the growth curves shown in Figure 1, of *T. suecica* , *D. salina* , *D. salina* 3×NaCl and *P. tricornutum* grown in control (CTR) or S‐limiting (S‐lim) conditions. Max cell density indicates the cell concentration at day 11 of the growth curves shown in Figure 1. Cells sampled during the mid‐late exponential growth phase were also characterized for their dry weight (pg cell^−1^), cell volume (fl), macroelemental (C, N, S and P) cell quota expressed as a percentage of the cell's dry weight (%C, %N, %S, %P) and C stable isotope fractionation (δ^13^C). For all parameters values are expressed as mean ± standard deviation of at least three independent replicates. Asterisks indicate a significant difference between the S‐lim sample and the respective CTR sample (*t*‐test, *p < 0.05; **p < 0.01; ***p < 0.001).

In the case of *D. salina* , we tested the response to S‐lim in cells grown at two different salinities: the standard salinity of the growth medium (hereafter CTR and S‐lim samples, Figure 1B) and a growth medium with a 3‐fold NaCl concentration, usually employed to cultivate this halophilic species (hereafter 3×NaCl CTR and 3×NaCl S‐lim, Figure 1C). Both CTR and 3×NaCl CTR *D. salina* cultures grew exponentially for about 1 week, then entered the stationary phase, reaching a higher final cell yield in the 3×NaCl CTR (Figure 1B,C, Table 1). S‐lim and 3×NaCl S‐lim *D. salina* cultures grew slower than the respective CTRs, as attested by both the lower cell counts and growth rates (Figure 1B,C, Table 1). However, during the second week of growth, the S‐lim cultures behaved differently: at lower salinity, S‐lim cultures recovered and reached a cell density slightly higher than that of the CTR (Figure 1B). Conversely, the maximum cell concentration in 3×NaCl S‐lim cultures stayed lower compared to the respective 3×NaCl CTR throughout the whole growth curve and did not reach the stationary phase in the time frame considered (Figure 1C), differently from all the other *D. salina* cultures (Figure 1B,C, Table 1).

In the case of the diatom *P. tricornutum* (Figure 1D), the reduced sulfate availability also impacted both the exponential and the stationary growth phases. In this case, S‐lim cultures showed a reduced growth rate and entered the stationary phase earlier compared to the CTR, as indicated by a rather constant cell density in the S‐lim sample from day 4 on, which was less than half the maximum cell density of the CTR (Figure 1D, Table 1).

Since the sulfate concentration of 50 μM limited the growth of microalgae by reducing the growth rate and/or by affecting the final cell density, but not too severely (Figure 1 and Table 1), it was used to investigate how acclimation to S‐limitation modulated cell composition and photosynthesis in each species. All the following analyses were performed on cells harvested from the mid‐late exponential growth phase.

### 3.2 The Macroelemental and Macromolecular Composition of Microalgal Cells

S‐limitation influenced cell dry weight (DW) differently based on the species. In *T. suecica*, cell DW was roughly the same in both CTR and S‐lim samples. In *D. salina* , the salinity of the medium impacted the effects of S‐limitation, with S‐lim cells showing a higher DW with respect to the CTR cells only when grown at 3×NaCl conditions. In *P. tricornutum* , instead, the change of cell DW was particularly evident, with S‐lim grown cells having a DW about 3.6 times higher than that of the CTR (Table 1).

The cell quota of the macroelements C, N, S, and P was also quantified by means of CHN elemental analysis and TXRF spectroscopy. In both green microalgae, C represented almost 50% of cell DW in cells grown in CTR conditions, while in *P. tricornutum* , the C cell quota was slightly higher, reaching about 52% (Table 1). The cell quota of N ranged between about 3% in *D. salina* CTR to about 8% in *P. tricornutum* ; %S was about 0.5% or lower, and %P ranged from less than 0.1% in *D. salina* 3×NaCl CTR to about 0.3% in the diatom (Table 1). S‐limitation induced modulation of the cellular amount of C, N, S, and P was species‐specific (Table 1). In detail, the cell quota of C and S were unaffected by S‐limitation in *T. suecica* , while the %N and the %P increased in S‐lim cells. In *D. salina* , instead, %C significantly decreased both in S‐lim and 3×NaCl S‐lim cells, as did the %S. N and P showed distinctive changes according to the salinity of the medium. %N was roughly stable among CTR and S‐lim samples, while 3×NaCl S‐lim cells displayed a lower %N compared to 3×NaCl CTR. On the contrary, %P was decreased only in S‐lim cells. In the diatom *P. tricornutum*, S‐lim sample showed a significant change only in the cell quota of S, which was lower compared to the CTR.

We also analysed the C stable isotope composition, a parameter that depends on the C stable isotope fractionation during the various enzymatic reactions of C metabolism, starting from C fixation. On average, CTR cells had a δ^13^C value of about −15 in *T. suecica* , −14 in *D. salina*, and −24 in *P. tricornutum* . In all species, S‐lim cells showed δ^13^C values significantly lower (i.e., more negative) than the respective CTR samples (Table 1), suggesting changes in the C metabolism upon S‐limitation.

**FIGURE 2:**
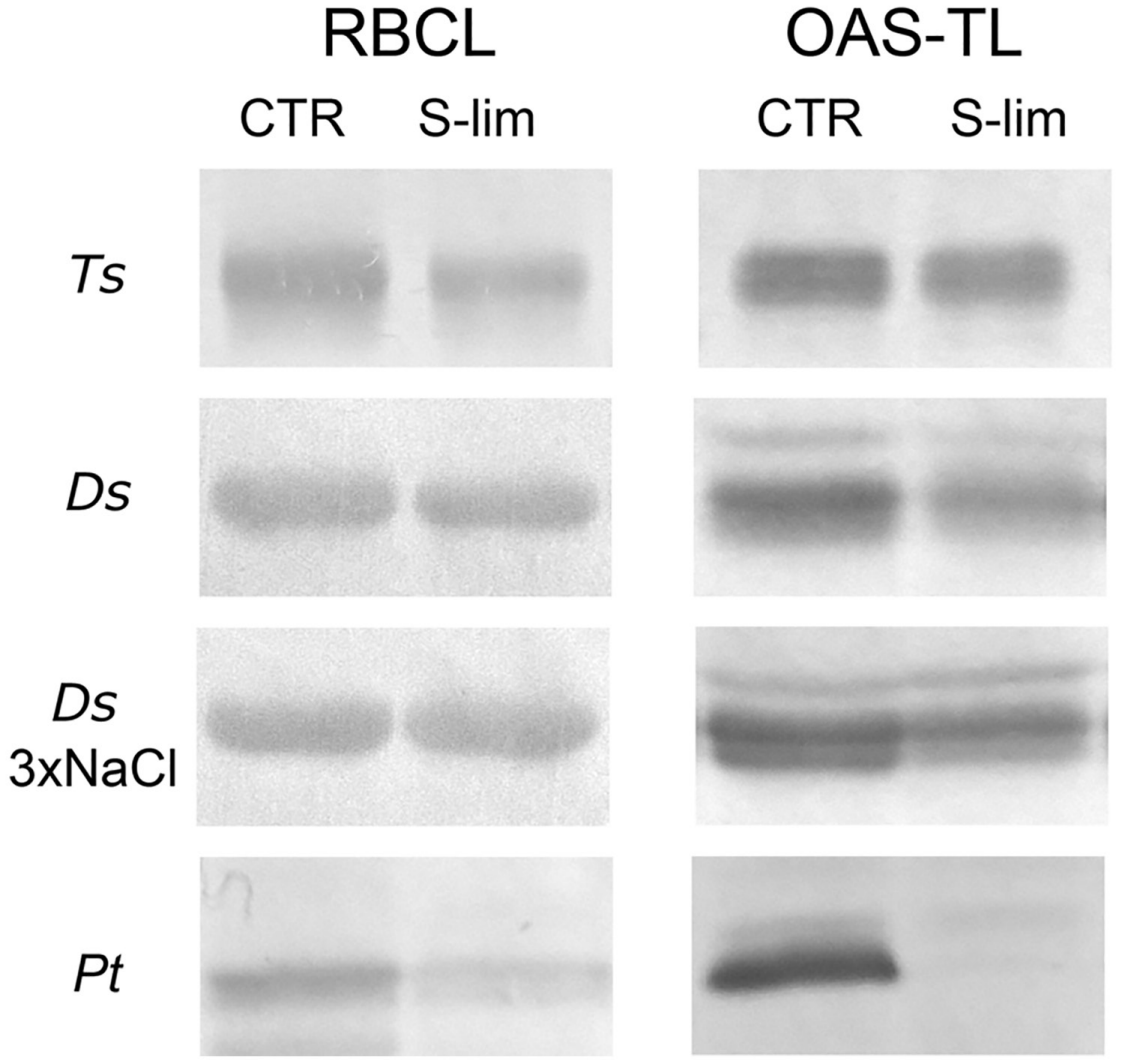
Effect of S‐limitation on the RBCL and OAS‐TL content in microalgae cells. Immunoblot analysis of total protein extracts of *T. suecica* (Ts), *D. salina* (Ds), *D. salina* 3×NaCl (Ds 3×NaCl) and *P. tricornutum* (Pt), grown in control (CTR) or S‐limiting (S‐lim) conditions using antibodies against RuBisCO large subunit (RBCL) and O‐acetylserine (thiol) Lyase (OAS‐TL). On the SDS‐PAGE used for western blotting different amounts of Chl were loaded per lane depending on species and antibody, keeping constant the amount of Chl loaded for each S‐lim sample and the respective CTR. In detail, the following amounts of Chl were loaded: For anti‐RBCL, 0.5 μg Chl for *T. suecica* and *D. salina* and 1 μg Chl for *P. tricornutum* ; for anti‐OAS‐TL A, 1 μg Chl for all species.

To gain additional insights into macroelement assimilation, we tested whether the accumulation of key enzymes involved in C and S assimilation, that is, RuBisCO and O‐acetylserine(thiol)Lyase (OAS‐TL), respectively, was affected in cells grown under S‐limitation. By performing immunoblot analysis on total cellular protein extracts, previously separated on SDS‐PAGEs (Figure S1), we found that in the green microalgae, the band immunodetected by the antibody raised against RuBisCO large subunit (RBCL) was rather similar in CTR and S‐lim samples, whereas it was reduced in *P. tricornutum* S‐lim compared to the CTR (Figure 2, Figure S2). In the case of OAS‐TL immunodetection, one main band was recognized by the anti‐OAS‐TL A antibody in all species, together with other faint bands in the case of *D. salina* and the diatom. This is in line with data available for other species, showing that multiple isoforms of the enzymes are present both in plants and in algae (Heeg et al. 2008; Gonzalez‐Ballester and Grossman 2009; Bromke et al. 2013; Carfagna et al. 2011). The effect of S‐limitation on the accumulation of the OAS‐TL enzyme was diversified depending on the species. The signal of anti‐OAS‐TL A immunodetected bands was rather similar between CTR and S‐lim samples in *T. suecica* , while it was fainter in S‐lim compared to the CTR in *D. salina* , irrespective of the salinity of the growth medium, and was markedly reduced in *P. tricornutum* , where the band was almost undetectable in S‐lim protein extracts (Figure 2, Figure S2). Overall, the immunoblot data suggest that OAS‐TL accumulation was modulated upon S‐limitation, but we cannot exclude other isoforms not recognized by the antibody employed, which would display a different dependence on the sulfate availability.

Changes in nutrient assimilation and in elemental composition are often paralleled by a modulation in the overall cellular macromolecular composition. In all tested samples, the amount of proteins per cell increased in S‐lim samples, but to a different extent according to the species (Table S1). *P. tricornutum* showed the highest increase, in line with the heavier S‐lim cells compared to CTR ones (Table 1, Table S1). The changes in the proteome, likely, were not only quantitative, as suggested by the Coomassie stained SDS‐PAGE profile of the total cellular protein extracts, in which differences in the intensity of some bands between S‐lim and CTR extracts were present, particularly in *D. salina* and *P. tricornutum* (Figure S1). The overall macromolecular cell composition of the three microalgae was also evaluated by Fourier Transformed Infrared spectroscopy (FTIR) analyses on whole cells, a protocol allowing the estimation of proteins, carbohydrates, lipids (and also silicates for *P. tricornutum* ) relative amounts in each sample based on specific band assignments of the functional groups of the various macromolecules (Giordano et al. 2001). After S‐lim acclimation, in all tested species at least one of the macromolecular pool ratios (lipids/proteins, carbohydrates/proteins, lipids/carbohydrates) was significantly different from the respective CTR, but with changes diversified according to the species (Table S1). In *T. suecica* , S‐limitation induced a significant decrease in the carbohydrate/protein ratios and an increase in the lipid/carbohydrate ratios. In *D. salina* and *P. tricornutum* , on the contrary, the carbohydrate/protein ratios were significantly higher in S‐lim cells compared to the respective CTR. Moreover, the 3×NaCl growth condition induced the highest modulation of the cell composition in *D. salina* , with all the ratios significantly different between CTR and S‐lim samples. In *P. tricornutum* , in addition to the carbohydrate/protein ratio, the Si/protein ratio also creased in S‐lim cells.

### 3.3 Microalgae Pigment Profile and Photosynthetic Activity

**FIGURE 3:**
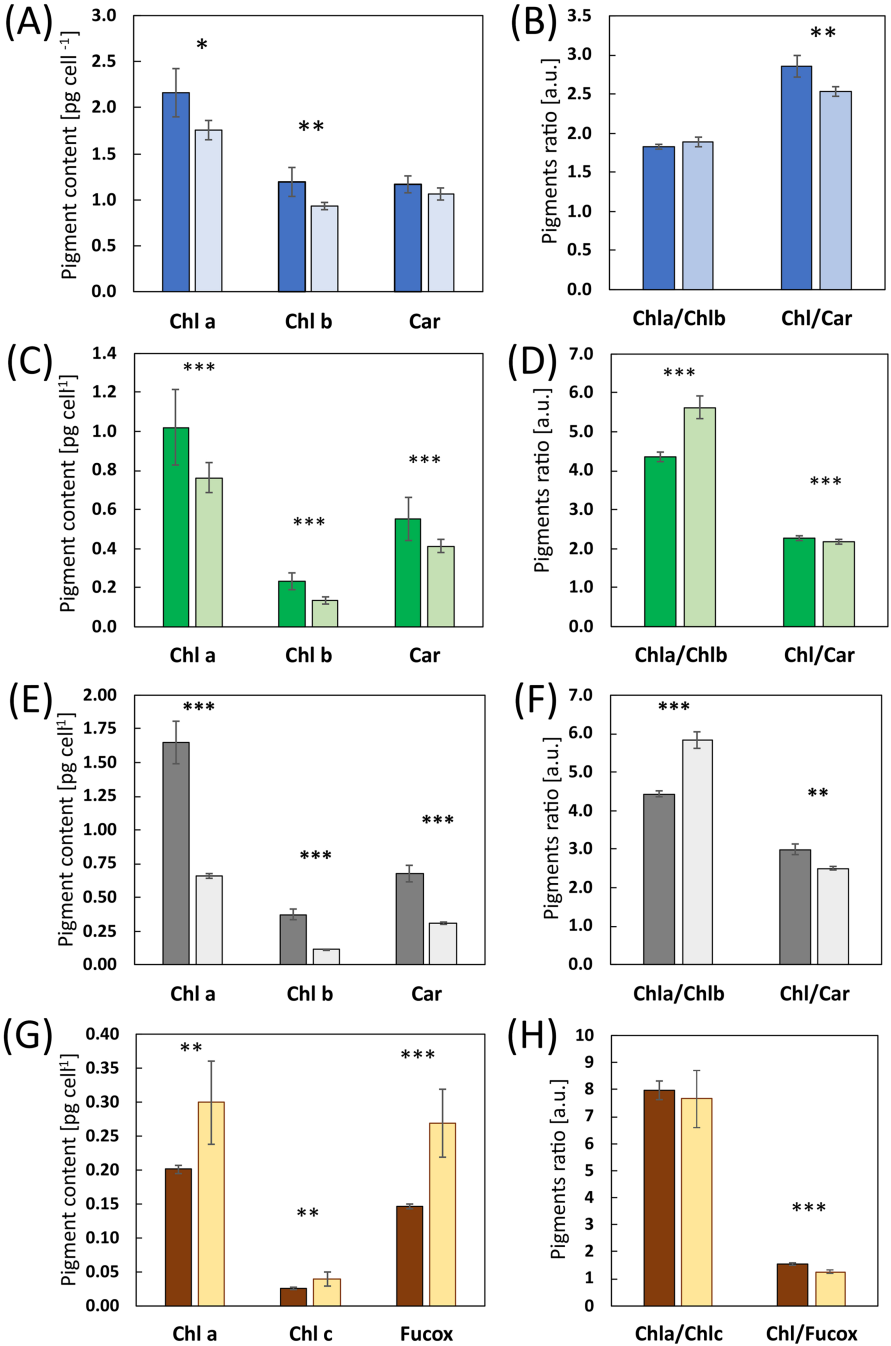
Photosynthetic pigment quantification in microalgal cells grown in control and S‐limiting conditions. Data for pigments quantification is shown in blue for *T. suecica* (A, B), in green for *D. salina* (C, D), in gray for *D. salina* 3×NaCl (E, F) and in orange for *P. tricornutum* (G, H). In all panels darker color indicates samples grown in control (CTR) conditions and lighter color S‐limited (S‐lim) samples. In panels A, C, E, and G the histograms show the cell quota of chlorophyll (Chl) *a*, accessory Chls (Chl *b* in *T. suecica* and *D. salina* , Chl *c* in *P. tricornutum* ) and total carotenoids (Car) in *T. suecica* and *D. salina* , or fucoxanthin (Fucox) in *P. tricornutum* , expressed as pg cell^−1^. In panels B, D, F, and H the histograms show the corresponding ratio of Chl *a*/accessory Chl and total Chl/Car or Chl/Fucox in case of *P. tricornutum* , expressed as arbitrary units (a.u.). Data are shown as mean ± standard deviation of at least three independent replicates and asterisks indicate a significant difference between the S‐lim sample and the respective CTR sample (*t*‐test, *p < 0.05; **p < 0.01; ***p < 0.001).

In order to evaluate the effects of S‐limitation on photosynthesis, we characterized microalgal cells both biochemically and in vivo by biophysical methods. In both green microalgae, the accumulation of Chl *a* and Chl *b* per cell decreased after S‐limitation (Figure 3A,C,E). *T. suecica* and *D. salina* instead differed for the changes in the Chl *a*/*b* ratio, which was rather constant and equal to around 1.8 in *T. suecica* (Figure 3B). *D. salina* , irrespective of salinity, increased the Chl *a*/*b* ratio in S‐lim samples, from about 4.3 of both CTR samples to 5.6 of S‐lim cells (Figure 3D,F). The two green microalgae also decreased the Chl/Car ratio indicating that total carotenoids in S‐lim cells were accumulated to a greater extent on a per Chl basis, despite the overall Car reduction in *D. salina* (Figure 3C,E). The diatom *P. tricornutum* displayed an opposite trend, showing a higher accumulation of photosynthetic pigments per cell in S‐lim conditions (Figure 3G). However, as described above, *P. tricornutum* displayed a huge increase in DW when grown in S‐limitation (Table 1). Thus, it still appeared to have about half the pigment content per biomass unit, as Chl *a* accounted for 1.9% of cell DW in CTR and 0.8% only in S‐lim cells, and also Chl c and Fucox decreased similarly. The Chl *a*/*c* ratio instead was not affected by sulfate concentration, while the Chl/Fucox ratio decreased after S‐lim acclimation (Figure 3H). Pigment extracts were also analysed through HPLC, to verify whether the changes in overall Car content per cell detected spectrophotometrically were paralleled by changes in the Car profile. In *T. suecica* this was not the case, as both the Car content per cell (Figure 3) and the Car profile of CTR and S‐lim cells were almost unchanged (Table 2). On the contrary, *D. salina* and *P. tricornutum* modulated both the absolute amount per cell and the Car profile (Figure 3, Table 2). In *D. salina* , in the cultures grown at lower salinity, the relative content of β‐γ‐car, an intermediate of Car biosynthesis, and of lutein was increased in S‐lim cells compared to the CTR, while the xanthophyll cycle pigments violaxanthin and antheraxanthin showed an opposite trend (Table 2). In samples grown at 3×NaCl, only β‐γ‐car showed a significant change, being more abundant in S‐lim cells (Table 2). In *P. tricornutum* , HPLC analyses revealed a relative increase in the xanthophyll cycle pigments diadinoxanthin and diatoxanthin in S‐lim with a parallel reduction of fucoxanthin (Table 2).

**TABLE 2:** Carotenoid profile of *T. suecica* , *D. salina* , *D. salina* 3×NaCl, and *P. tricornutum* .

		CTR	S‐lim
*T. suecica*	9‐cis neoxanthin	13.2 ± 5.3	10.3 ± 3.3
	violaxanthin	21.5 ± 2.1	20.5 ± 0.6
	lutein	27.7 ± 3.7	31.1 ± 1.7
	loroxanthin dodecenoate	7.1 ± 1.1	5.9 ± 0.4
	β‐γ‐car	15.1 ± 3.6	16.3 ± 2.0
	β‐ε‐car	3.3 ± 1.1	2.0 ± 0.3
	β‐β‐car	12.1 ± 0.5	14.0 ± 0.4**

*D. salina*	9‐cis neoxanthin	7.3 ± 3.6	4.4 ± 1.3
	violaxanthin	19.4 ± 2.4	11.5 ± 1.8**
	antheraxanthin	5.1 ± 0.6	1.6 ± 0.8**
	lutein	43.6 ± 2.9	50.2 ± 1.5*
	zeaxanthin	2.4 ± 0.5	4.0 ± 2.6
	β‐ γ‐car	1.7 ± 0.4	5.9 ± 0.2***
	β‐β‐car	20.5 ± 1.8	22.3 ± 2.9

*D. salina* 3×NaCl	9‐cis neoxanthin	5.5 ± 1.8	4.8 ± 0.5
	violaxanthin	11.9 ± 2.7	12.8 ± 0.5
	antheraxanthin	6.8 ± 3.0	4.1 ± 0.8
	lutein	46.2 ± 3.7	47.5 ± 1.6
	zeaxanthin	5.6 ± 1.8	4.1 ± 3.9
	β‐γ‐car	2.5 ± 0.3	6.0 ± 0.8**
	β‐β‐car	21.5 ± 2.1	20.7 ± 2.2

*P. tricornutum*	fucoxanthin	67.5 ± 2.8	54.5 ± 3.8**
	diadinoxanthin	25.5 ± 2.9	37.9 ± 3.6**
	diatoxanthin	0.5 ± 0.2	1.6 ± 0.3**
	β‐β‐car	6.5 ± 0.4	6.0 ± 0.2

*Note:* The table shows the relative abundances of each carotenoid identified by HPLC analyses in *T. suecica* , *D. salina* , *D. salina* 3×NaCl, and *P. tricornutum* samples, calculated from the area of its peak in the chromatogram over the total areas of all identified carotenoid peaks. Data are shown as mean ± standard deviation of at least 3 independent replicates, and asterisks indicate a significant difference between the S‐lim sample and the respective CTR sample (*t*‐test, *p < 0.05; **p < 0.01; ***p < 0.001).

**FIGURE 4:**
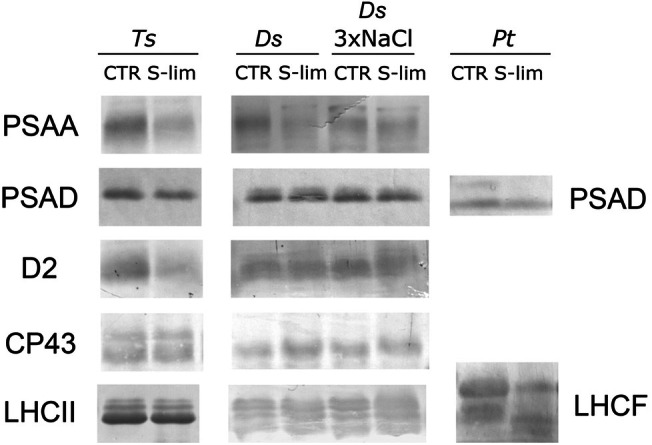
Effect of sulfur limitation on the content of main subunits of the photosynthetic apparatus in microalgae cells. Immunoblot analysis of total protein extracts of *T. suecica, D. salina, D. salina* 3×NaCl and *P. tricornutum* , grown in control (CTR) or S‐limiting (S‐lim) conditions using antibodies against the PSI subunits PSAA and PSAD, the PSII subunits CP43 and D2, and the antenna proteins LHCII and LHCF. On the SDS‐PAGE used for western blotting different amounts of Chl were loaded per lane depending on species and antibody, keeping the amount of Chl loaded for each S‐lim sample and the respective CTR sample constant. In detail, the following amounts of Chl were loaded: For *T. suecica* , 1.2 μg for PSAA and D2, 0.5 μg for PSAD, 0.4 μg for CP43 and LHCII; for *D. salina* , 0.15 μg for PSAA, 0.5 μg for PSAD, 0.3 μg for CP43 and LHCII and 0.4 μg for D2; for *P. tricornutum* , 1 μg for PSAD and LHCF.

The effects of S‐limitation on the photosynthetic apparatus were also investigated by performing SDS‐PAGE on total cellular protein extracts (Figure S1) and immunoblotting, using available antibodies against different thylakoid membrane proteins, namely the PSI subunits PSAA and PSAD, the PSII core proteins CP43 and D2, and the antenna proteins LHCII and LHCF (Figure 4). Relative changes in protein abundance in response to S‐limitation were estimated by densitometry on immunodetected bands (Figure S2).

After S‐limitation, the amount of the PSI and PSII core subunits showed a tendency to decrease in *T. suecica* , whereas this change was negligible in *D. salina* . In both green algae, the amount of LHCII antennae, immunodetected as multiple bands, was almost unchanged in the different growth conditions (Figure 4). However, it is worth mentioning that, as we were employing antibodies raised against plant isoforms, particularly in the case of antenna proteins, we might not have been able to detect all LHCII isoforms in the two microalgae, as shown previously in *C. reinhardtii* (Girolomoni et al. 2017), thus possibly missing detecting changes in some specific subunits. Indeed, on the Coomassie stained SDS‐PAGE, *D. salina* displayed a band with an apparent molecular weight compatible with that of LHCII proteins, showing a different intensity in CTR and S‐lim samples, which however was likely not detected by the anti‐LHCII antibody used on the corresponding immunoblotting (Figure S3). In the case of *P. tricornutum* , the antibody against the antennae LHCF detected two main bands in the CTR sample. The intensity of both bands was modulated in the S‐lim sample, with the upper one strongly reduced, while the lower one was more intense compared to the CTR, showing an overall reduction of the amount of LHCF in the S‐lim sample. The amount of the PSI PSAD subunit also decreased in *P. tricornutum* after S‐limitation (Figure 4, Figure S2).

Photosynthesis was also assessed in vivo by exploiting PAM fluorometry coupled with P700^+^ absorption signal analyses to verify whether the S‐limitation had an impact on photosynthetic light reactions. The maximum PSII quantum yield, Fv/Fm, showed a significant reduction after S‐lim acclimation only in *D. salina* 3×NaCl and in *P. tricornutum* (Table S2). Pm, the maximum P700^+^ signal, decreased in all S‐lim samples except in *P. tricornutum* (Table S2). As the Pm signal is proportional to the amount of oxidizable P700 present in the sample, the reduction of Pm observed is in line with the decrease in Chl *a* per cell detected in *T. suecica* and *D. salina* 3×NaCl (Figure 3A,E, Table S2). In the case of *D. salina* and *P. tricornutum* , instead, the change in Chl *a* per cell did not fully match the changes in Pm (Figure 3C,G, Table S2). In fact, under S‐limitation, *D. salina* cells showed roughly 75% the Chl *a* content of the CTR and a Pm almost halved, whereas *P. tricornutum* cells had a 1.5‐fold increase in Chl *a* content and a Pm almost unchanged. The discrepancy between the fold changes of Chl *a* per cell and of Pm signal can be due to a reduced PSI content, as suggested by anti‐PSAD immunoblotting in *P. tricornutum* (Figure 4), and/or to a partial inactivation of PSI in S‐lim cells.

**FIGURE 5:**
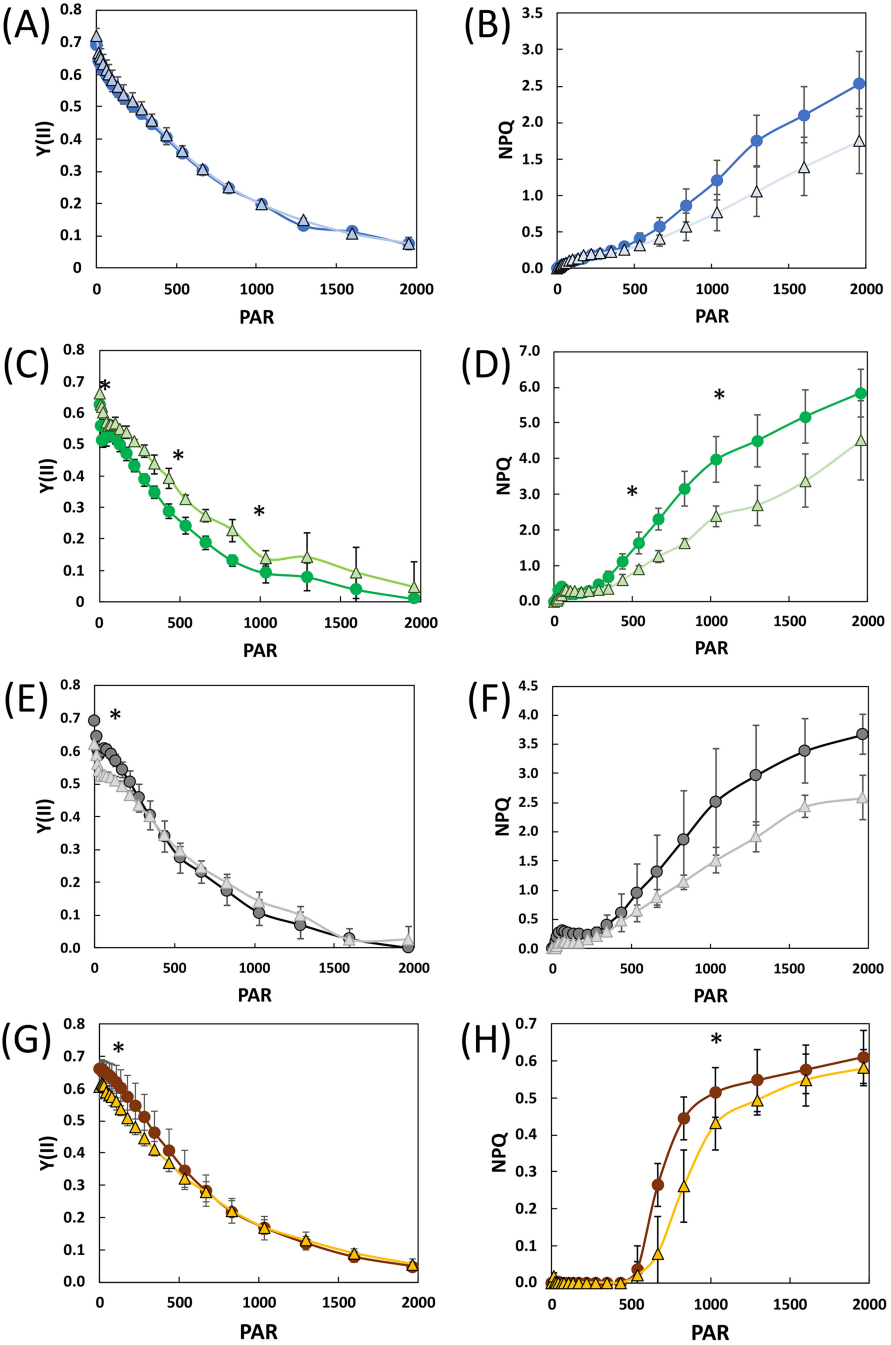
Chlorophyll fluorescence analyses of *T. suecica, D. salina* and *P. tricornutum* . The graphs show the Chlorophyll fluorescence derived parameters PSII quantum yield (Y[II]) and non‐photochemical quenching (NPQ) of *T. suecica* in blue (A, B), *D. salina* in green (C, D), *D. salina* 3×NaCl in gray (E, F) and *P. tricornutum* in orange (G, H) samples grown in control (CTR) or S‐limited (S‐lim) conditions. In all panels, the CTR sample is shown in circles and darker color, S‐lim in triangles with lighter color. PAR: Photosynthetic active radiation (μmol photons m^−2^ s^−1^). The data are shown as mean ± standard deviation of at least three independent light curve kinetics. Statistical significance is shown only for light curves steps at 100, 530, and 1000 μmol photons m^−2^ s^−1^ (*t*‐test, *p < 0.05).

In all species, the effective quantum yields of PSII and PSI, that is, Y(II) (Figure 5) and Y(I) (Figure S4), respectively, declined as the cells were exposed to increasing actinic light intensity, as expected. NPQ, the Chl‐fluorescence derived parameter associated with photoprotection, was instead induced as the irradiation got higher, in line with the common response of photosynthetic cells, which activate photoprotection mechanisms to face strong light. The three species tested, however, differed for the specific kinetics of NPQ upon light curve exposure. *T. suecica* showed a roughly linear increase of NPQ as light increased (Figure 5B), *D. salina* kept low NPQ values till around 400 μmol photons m^−2^ s^−1^, then it increased till 2000 μmol photons m^−2^ s^−1^. *P. tricornutum* showed negligible NPQ up to about 500 μmol photons m^−2^ s^−1^, followed by a sudden activation of NPQ which saturated at around 1000 μmol photons m^−2^ s^−1^ (Figure 5H). The maximal NPQ values were also species‐specific and quite different between the green algae and the diatom.

When considering all the measured photosynthetic parameters together, it is evident that S‐limitation had a diversified impact on the photosynthetic response of the analysed microalgae. S‐limitation did not affect significantly the photosynthetic processes in *T. suecica* (Figure 5A,B), whereas it exerted a different effect depending on the salinity of the growth medium in *D. salina* . In the standard medium, S‐lim cells displayed an even higher Y(II) (Figure 5C) and a lower NPQ (Figure 5D) compared to the CTR sample, while 3×NaCl S‐lim cells, in addition to the reduced Fv/Fm (Table S2) also showed a reduced Y(II) at low actinic light intensities (Figure 5E) compared to the 3×NaCl CTR. Similarly, in *P. tricornutum* , Y(II) was reduced in the first steps of the light curve in S‐lim cells (Figure 5G). Moreover, S‐limitation affected NPQ activation of this diatom, since S‐lim cells required a stronger light intensity to induce NPQ, despite reaching the same maximal amplitude of the CTR (Figure 5H). Despite the differences detected in PSII‐related parameters and in Pm (Figure 5 and Table S2), the Y(I) did not show major differences in S‐lim cells in any of the species tested (Figure S4).

## 4 Discussion

### 4.1 Under S‐Limitation, Resources Are Allocated Primarily to Photosynthesis

Macronutrient limitation usually affects cell division and biomass production in microalgae (Huysman et al. 2010; Markou et al. 2014). Although the specific effects may vary based on both the limiting nutrient and the algal species, reduced growth occurs due to an energy deficit. Resources, in fact, may be redirected to increase nutrient uptake (Camargo et al. 2007; Takahashi et al. 2011; Sanz‐Luque et al. 2015) and cell metabolism is generally shifted to the synthesis of storage compounds that do not require the limiting nutrient, such as starch and neutral lipids in the form of triacylglycerols when N is limiting (Simionato et al. 2013; Markou et al. 2014; Procházková et al. 2014). Photosynthesis is also often negatively affected, both in the light reactions and in C fixation (Berges et al. 1996; Zhang et al. 2004; Simionato et al. 2013; Procházková et al. 2014). This may easily lead to an over‐reduction of the photosynthetic electron transport chain, followed by ROS production and oxidative stress, which photosynthetic cells counteract by enhancing photoprotective mechanisms, such as NPQ and alternative electron transport pathways (Zhang et al. 2004; Simionato et al. 2013), and by accumulating antioxidant molecules such as carotenoids (Coesel et al. 2008). In the specific case of S limitation, previous studies in the freshwater algal species *C. reinhardtii* showed that, in the first 24 h after S starvation, transcription of genes encoding subunits involved in light harvesting and electron transport was reduced, opposite to others encoding proteins involved in photoprotection, that is, regulating the flow of light energy (LHCSR) or electrons (PTOX) in the photosynthetic light reactions, which were induced. In *C. reinhardtii* , many of these changes were shown to be mediated by the SAC1 protein (Davies et al. 1996; Zhang et al. 2004; Pollock et al. 2005; Gonzalez‐Ballester and Grossman 2009; Takahashi et al. 2011). Such studies evidenced the mutual dependence of photosynthesis and S, with the former requiring S for proper functioning while providing the metabolic energy supporting S assimilation (Takahashi et al. 2011; Giordano and Raven 2014). However, previous studies mostly focused on acute S starvation, while the effects of continuous low S availability and on marine microalgae are limited. This is most likely because S is not a limiting factor for algal growth in today's oceans. Yet, evidence from biogeochemical, paleontological, and ecological studies suggests that changes in oceanic S availability may have influenced phytoplankton abundances throughout Earth's history (Ratti et al. 2011). Moreover, due to the high energy requirement for its assimilation (Giordano and Raven 2014), changes in other environmental parameters that elicit an energy demanding response by algae are likely to impact sulfur assimilation even under current conditions (Ferrari et al. 2022).

This idea is well in line with the behavior we detected in *D. salina* , in which the concomitant occurrence of S‐limitation and high salinity had a stronger impact on growth (Figure 1), cell composition (Table 1, Table S1) and photosynthetic efficiency (Figure 5, Table S2) with respect to the sole S‐limitation, despite sulfate uptake in *D. salina* being shown to be specifically dependent on Na^+^‐symport mechanisms (Weiss et al. 2001). Indeed, *D. salina* S‐lim 3×NaCl cells had to face a combined stress condition in which they had to adjust osmolarity and optimize the usage efficiency of available S and resources as well. Among the differences detected in the elemental composition, 3×NaCl CTR samples showed a higher N cell quota compared to all other *D. salina* samples (Table 1). This could be due, at least to some extent, to the accumulation of the quaternary ammonium compound glycine betaine, acting as an osmolyte in response to high salinity, as shown in other studies (Mishra et al. 2008). 3×NaCl S‐lim cells, instead, failed to accumulate the high amount of cellular N observed in the respective 3×NaCl CTR, suggesting that S‐limitation hampers their ability to modulate cell physiology to respond to both high salinity and S‐limitation. This is possibly related to the effects of S‐limitation on photosynthesis. When comparing the growth and photosynthetic response of CTR and S‐lim cells (Figure 1, Figure 5), indeed, it clearly emerges that the S‐lim samples with the most affected photosynthetic performance (i.e., 3×NaCl *D. salina* and *P. tricornutum* ) also displayed the major growth impairment.

All species, however, showed differences between CTR and S‐lim samples when the photosynthetic apparatus was characterized biochemically. We detected a decrease in chlorophyll accumulation after S‐lim acclimation (Figure 3), which was likely the result of an acclimation process allowing the microalgae to optimize their photosynthesis by adjusting the stoichiometry of their photosynthetic apparatus subunits, as exemplified by the changes in PS core subunits in *T. suecica* and antenna proteins in *P. tricornutum* (Figure 4, Figure S2). All species showed changes in the accumulation of Car per Chl molecules (Figure 3) and enhanced accumulation of photoprotective Car, like lutein in *D. salina* and xanthophyll cycle pigments in *P. tricornutum* (Table 2; Jahns and Holzwarth 2012; Goss and Latowski 2020). Notably, despite the common trend in modulating the accumulation of pigments and protein subunits of the photosynthetic apparatus, the specific changes detected at the molecular level diversified from species to species and are likely due to the diversity in the photosynthetic apparatus itself among species rather than a consequence of a different effect of S‐limitation on it. Besides the well‐known differences between green microalgae and the model diatom *P. tricornutum* in terms of pigments and photosynthetic apparatus composition (Koziol et al. 2007; Wilhelm et al. 2014; Erickson et al. 2015; Büchel 2020), also the two analysed green microalgae *T. suecica* and *D. salina* displayed different features. In CTR conditions, *T. suecica* cells displayed more Chls per cell and a lower Chl *a*/*b* ratio with respect to *D. salina* (Figure 3). They also differ in their Car profile, with only *T. suecica* accumulating loroxanthin (Table 2), a pigment typical of some green algae and particularly abundant in low light conditions (Garrido et al. 2009; Sansone et al. 2017; Di Lena et al. 2019; van den Berg and Croce 2022). Overall, such features suggest that *T. suecica* is enriched in antenna proteins with respect to *D. salina*, which indeed displayed a Chl *a*/*b* ratio close to values considered a sign of a small antenna size (Ware et al. 2020). Changes in the NPQ kinetics were also highly species‐specific, both considering its activation and the amplitude, in line with the well‐known biodiversity of this photoprotection mechanism, especially in algae (Wilhelm et al. 2014; Erickson et al. 2015; Goss and Lepetit 2015; Lacour et al. 2019). While *T. suecica* has been poorly characterized for its photosynthesis, *D. salina* is a common model species to study adaptations to high salinity and has been shown to harbor unique adaptations in its photosynthetic apparatus, like a “mini‐PSI” composed of a reduced number of core subunits, a configuration suggested to enable the species to accumulate more PSI, thereby better supporting the accumulation of osmolytes in high salinity (Caspy et al. 2020), and specific antenna subunits responsive to nutrient limitations, such as the TIDI1 antenna protein, which accumulates in response to low Fe availability (Davidi et al. 2023). Although we still lack detailed knowledge at a molecular level on the changes induced by S‐limitation in the photosynthetic apparatus, the data collected in this work strongly suggest that the species we analysed activated specific responses to optimize photosynthetic light reactions under S‐limitation.

Irrespective of the specific composition of the photosynthetic apparatus and the changes induced by S‐limitation on specific pigments and photosynthetic subunits, all species appeared to prioritize the allocation of available resources to maintain the photosynthetic efficiency unchanged or close to that of cells grown in nutrient‐replete conditions. This, however, came at the expense of growth and was also accompanied by changes in cell composition in S‐lim cells.

Reduction in growth may result from multiple factors, starting from S scarcity itself, which makes S the resource limiting the total amount of biomass in the culture. We recognized two distinct behaviors to face S limitation with respect to cell elemental composition in the analysed species (Table 1). *T. suecica* S‐lim cells, being unaffected in S and C cell quota compared to CTR (Table 1), showed a more homeostatic behavior (Giordano 2013). Hence, when S became less available during the batch culture growth, *T. suecica* S‐lim cells failed to further divide, and the culture reached the stationary phase earlier than the CTR (Figure 1). On the opposite, *D. salina* and *P. tricornutum* reduced the %S on DW (Table 1), minimizing the cellular requirement at least to some extent. This can be the consequence of an active regulation of cellular components, for example, via substitution of some proteins with “low‐S” variants, as shown in cyanobacteria for the phycobilisomes (Mazel and Marlière 1989; Giordano et al. 2015), or in the case of cell wall polypeptides in *C. reinhardtii* (Takahashi et al. 2001). Another reason for the reduced S cellular content may instead be an altered S assimilation, as both *D. salina* and particularly *P. tricornutum* appear to decrease the accumulation of OAS‐TL (Figure 2), an enzyme involved in Cys synthesis. However, we cannot exclude that either the decrease in OAS‐TL is a consequence of the lack of resources to accumulate as much enzyme as the CTR cells, or that other OAS‐TL isoforms, not detected by the antibody we employed here, may be overexpressed in S‐lim cells and complement the downregulation of the isoform we detected.

A second cause of growth reduction may be a shortage of metabolic energy. This would require the reallocation of available energy and resources among cellular functions and may impair the cell's ability to support both cellular maintenance and division. This idea fits with the changes in δ^13^C values observed in S‐lim cells compared to their respective CTR (Table 1). Bicarbonate is usually ^13^C enriched compared to CO_2_; thus, lower values of δ^13^C in S‐lim cells compared to those in CTR cells are related to a higher fractionation by RuBisCO and a possible lack or downregulation of carbon concentrating mechanisms (CCMs), as well as a lower contribution of the anaplerotic fixation to the biomass production (Giordano et al. 2000; Tcherkez et al. 2011). Indeed, this finding is in line with previous data in which *D. salina* grown under S‐limitation minimized the activity of PEP carboxylase (PEPC; Giordano et al. 2000). Reduced PEPC activity is often accompanied by a lower N content (Giordano et al. 2003), which was not detected here as, except for 3×NaCl S‐lim cells, %N on DW did not decrease in S‐lim samples compared to the respective CTR. The changes induced by S‐limitation on cell metabolism, thus, appear to be more wide‐ranging than the sole CCM and bicarbonate use, as suggested also by the modulation of macromolecular composition of samples (Table S1), and deserve future investigations to be fully clarified at the molecular level. Nevertheless, the metabolic shift likely allowed for the conservation of energy previously utilized on costly cellular processes such as active bicarbonate uptake in CCMs (Spalding 2008; Yamano et al. 2015; Tsuji et al. 2017), enabling its reallocation to prioritized processes, as already observed in microalgae (Petrucciani et al. 2022, 2023, 2024). In addition to the photosynthetic light reactions, RuBisCO also represents another core function in photosynthetic cells. Indeed, except for *P. tricornutum* , S‐lim cells maintained a steady accumulation of RuBisCO on a per Chl basis, that is, cells kept a balance between light reactions of photosynthesis and C fixation by RuBisCO. However, as in both green microalgae the Chl content per cell decreased, it is likely that also the absolute amount of RuBisCO was reduced. RuBisCO is known to be among the main cellular reservoirs of S and is a main sink for cellular energy, as also a considerable amount of N is required for the synthesis of RuBisCO itself and its regulatory proteins (Andersson 2008; Carmo‐Silva et al. 2015). It is thus not surprising that S‐lim cells tend to decrease the accumulation of this protein. Nonetheless, keeping the balance between light reactions and metabolic usage of ATP and reducing power is likewise essential for proper cell functioning, avoiding over‐reduction of the photosynthetic electron transport chain due to lack of metabolic consumption of its products, NADPH and ATP. Whether the reduced accumulation of RuBisCO in green algae acclimated to S‐lim is the cause of the reduction in Chls, or vice versa, needs to be elucidated in future experiments.

It is however striking that S‐lim cells, which based on the above are likely reducing CCMs, thoroughly modulating C metabolism and slightly reducing RuBisCO, kept the C cell quota almost unchanged. This is not the case if we also consider the reduced growth. During growth, the increase in C in the algal biomass, expressed as pg C ml^−1^ of culture, is lower in the S‐lim cells compared to the CTR (Table S3), implying that although the cells were able to keep the C cell quota close to that of nutrient replete media, C fixation was decreased, failing to sustain also cell duplication.

A further possible reason for an energy shortage in S‐lim grown cells is an increase in housekeeping processes. S‐limitation was previously shown to increase mistranslation of mRNA (Holland et al. 2010; Giordano et al. 2015). It was also previously shown that protein turnover decreases at lower growth rates (Quigg and Beardall 2003). Thus, in S‐lim cells, the combination of mRNA mistranslation and reduced protein turnover related to the lower growth rate may increase the retention time of dysfunctional proteins and, in part, explain the higher protein cell quota in S‐lim cells (Table S1). Protein turnover, already under optimal conditions, requires a considerable quota of metabolic energy (Quigg and Beardall 2003), which would be even exacerbated in conditions like S‐lim. Such cellular maintenance costs would increase the necessity for unchanged photosynthetic efficiency and further contribute to explaining the lower growth under S‐limiting conditions.

Whether the changes in cell composition we detected are pivotal to the effective acclimation to S‐limitation or whether they are a consequence of the unbalanced nutrient availability and of the following reallocation of available nutrients and resources is still an open question that requires future research and in‐depth molecular analyses.

### 4.2 Is the Impact of S‐Limitation the Same in Algae of Different Phylogenies?

Although S‐limitation affected the growth, cell composition, and photosynthesis of all three algal species, the impact of S‐lim on the diatom cells appeared to be stronger than that on the two green microalgae. In our experimental setup, under S limitation, a huge increase in cell size and DW was particularly evident in the diatom in *P. tricornutum* compared to the green marine microalgae (Table 1). Despite not being unique to diatoms (Gorbi et al. 2007; Marieschi et al. 2015), this evidence suggests that in *P. tricornutum* , cell division was strongly impaired under S‐limitation. Although not yet studied specifically in the case of S‐limitation, this is in line with the effects of other nutrient deprivation, shown to alter the cell cycle of *P. tricornutum* by modulating the expression of specific cyclins (Huysman et al. 2010). Under S‐limitation, *P. tricornutum* was also heavily affected in the accumulation of key enzymes involved in the assimilation of S (i.e., OAS‐TL, Figure 2) and increased the accumulation of pigments involved in the photoprotection mechanism (i.e., the xanthophyll pigments diadinoxanthin and diatoxanthin, Table 2). The photoprotective functions of diatoxanthin are multiple and include both fast mechanisms like NPQ and ROS scavenging in the thylakoid membranes (Goss and Latowski 2020). This suggests that *P. tricornutum* S‐lim cells are more prone to oxidative stress, possibly due to the over‐reduction of the electron transport chain, which can arise when metabolic sinks like C fixation and S assimilation are downregulated (Erickson et al. 2015).

Diatoms are secondary endosymbiotic algae belonging to the red lineage. Thus, *P. tricornutum* is phylogenetically quite distant from the two Chlorophyta *T. suecica* and *D. salina* , also analysed in this study (Falkowski et al. 2004; Archibald 2009). Although we cannot exclude that the differences we detected in S‐limiting conditions are due only to a species‐specific response, the more severe effects S‐limitation had on *P. tricornutum* compared to the two Chlorophyta can be evaluated also in the light of the so‐called “Sulphate facilitation hypothesis” (Ratti et al. 2011; Giordano et al. 2018). This hypothesis suggests that the oceans' increase in sulfate availability contributed to the dominance of the red algal lineage in the present oceans, in contrast to more ancient ones in which species belonging to the green lineage were the most abundant and sulfate availability was lower than today (Ratti et al. 2011; Giordano et al. 2018). This sulfur facilitation hypothesis is in line with the physiological changes we observed in microalgal species in the tested conditions, one of which mimics the Mesozoic, and modern, high‐sulfur conditions (i.e., 25 mM, CTR) while the other exacerbated the ancient low‐sulfur conditions (i.e., 50 μM, S‐lim). These findings may provide insights into the molecular processes that regulate cellular responses to fluctuating sulfate availability.

## Author Contributions

C.G. designed the research. M.M., C.P., and M.B. performed the experiments. M.M., N.L.R., A.N., C.P., and C.G. analysed the data. M.M. and C.G. wrote the manuscript. All authors reviewed and edited the manuscript.

## Supporting information


**Data S1:** Supporting Information

## Data Availability

The data that support the findings of this study are available from the corresponding author upon reasonable request.
