# Supplementary material for: Effects of Sulfate Limitation on Photosynthesis and Cell Composition of Unicellular Marine Microalgae of Different Phylogenies
Source: Physiol Plant. 2025 Jul 17;177(4):e70401. doi: 10.1111/ppl.70401 (PMC12269360; doi:10.1111/ppl.70401)
Supplement: Supplementary file 1 — Data S1: Supporting Information [file PPL-177-e70401-s001.pdf]

SUPPORTING INFORMATION

Manuscript: **Effects of sulfate limitation on photosynthesis and cell composition of unicellular marine microalgae of different phylogenies**

Miles Minio, Mariano Battistuzzi, Alessandra Norici, Nicoletta La Rocca, Cristina Pagliano, Caterina Gerotto

Figure S1. Electrophoretic profile of the total cellular protein extracts.

The figure shows the Coomassie stained SDS-PAGE of the total protein extracts of *T. suecica*, *D. salina*, *D. salina* 3xNaCl and *P. tricornutum*, grown in control (CTR) or S-limiting (S-lim) conditions. For each species, the same amount of Chl was loaded on the gel for CTR and S-lim samples (1.2 µg Chl for *T. suecica* and *D. salina*, 0.25 µg Chl for *P. tricornutum*). Asterisks indicate protein bands that display a different intensity when comparing the S-lim sample with the respective CTR.

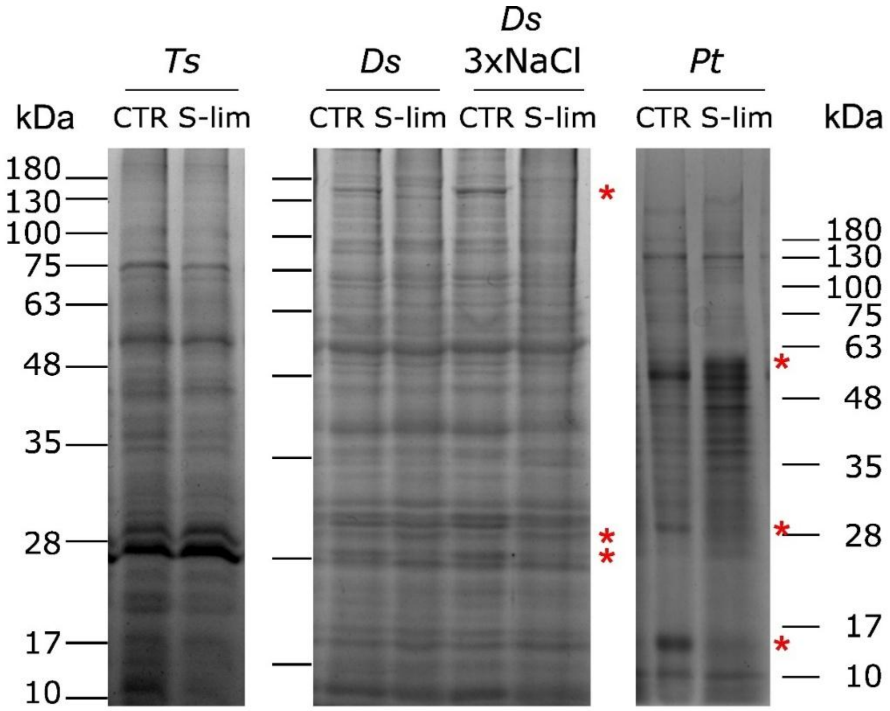

Figure S2. Densitometric analysis of immunoblotting.

The immunodetected bands shown in Figures 2 and 4 were analysed by using Quantity One software version 4.6.1 (Bio-Rad) for densitometry measurements. For each protein, the histograms represent relative ratios between intensities of S-lim/CTR samples, calculated as means ± standard deviations from at least three independent comparisons. Dashed lines indicate relative ratios of 0.8 and 1.2, used as cut-off values below and above which we considered a protein differently expressed in the S-lim sample with respect to the corresponding CTR sample.

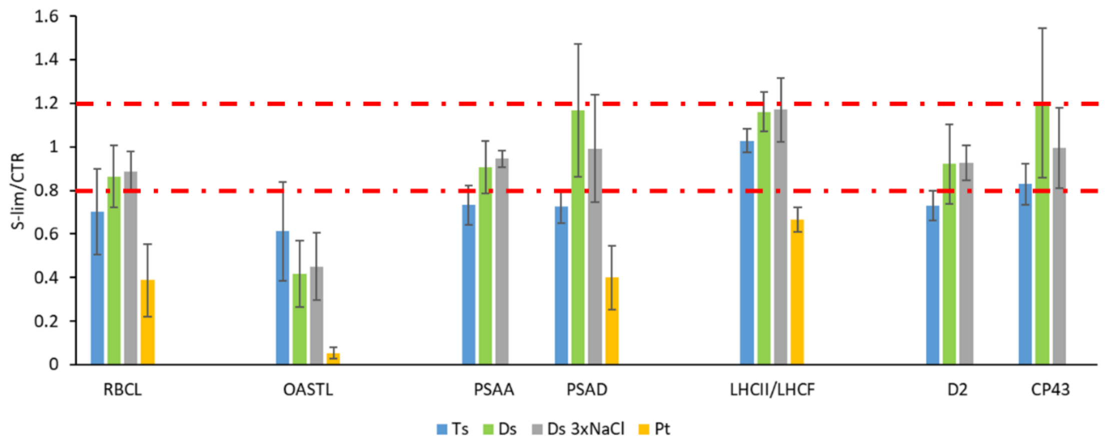

Figure S3. Comparison of the Coomassie stained SDS-PAGE portion in the 35-17 kDa range and the corresponding immunoblotting for the LHCII proteins in *D. salina*.

The band profile of the 35-17 kDa SDS-PAGE range of the Coomassie stained SDS-PAGE (A), and the multiple bands detected by the anti-LHCII antibody (B) were compared according to the positioning relative to the molecular weight markers. In both panels, asterisks indicate the Coomassie stained bands that are likely responsible for the anti-LHCII signal. The red arrow on the SDS-PAGE indicates a band showing a different intensity in CTR and S-lim samples in both Ds and Ds 3xNaCl samples in panel A, which appears not to be detected by the antibody on the corresponding immunoblotting (B).

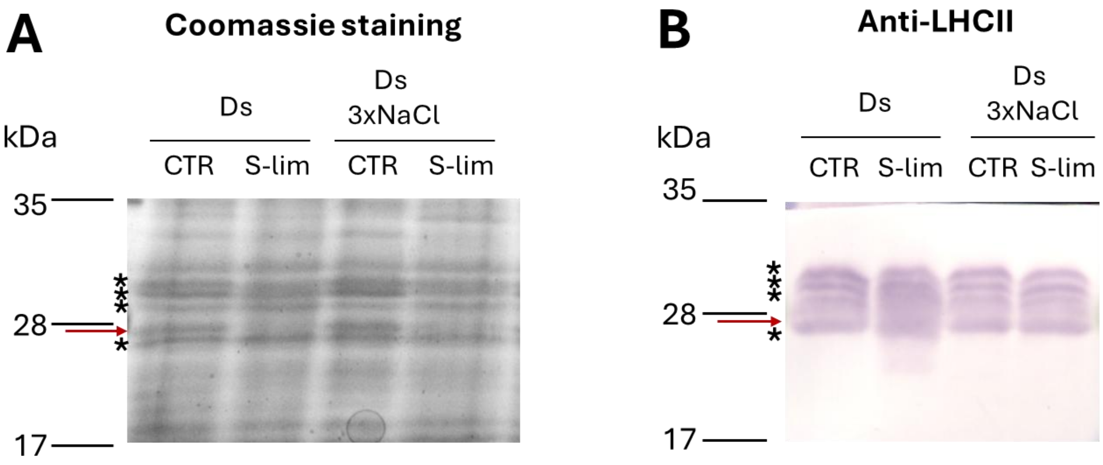

Figure S4. Photosystem I quantum yield Y(I) of microalgae grown in control and S-limited conditions.

The graphs show the PSI quantum yield [Y(I)] of *T. suecica* (A), *D. salina* (B), *D. salina* 3xNaCl (C) and *P. tricornutum* (D) samples grown in control (CTR) or S-limited (S-lim) conditions. In all panels, CTR culture is shown in circles and darker colour, S-lim culture in triangles with a lighter colour. The data are derived from P700+ absorption signal in light curves kinetics and are shown as mean ± standard deviation of at least 3 independent replicates. Statistical significance is shown only for light curves steps at 100, 530 and 1000 µmol photons m^{-2} s^{-1} (t test, * $p<0.05$).

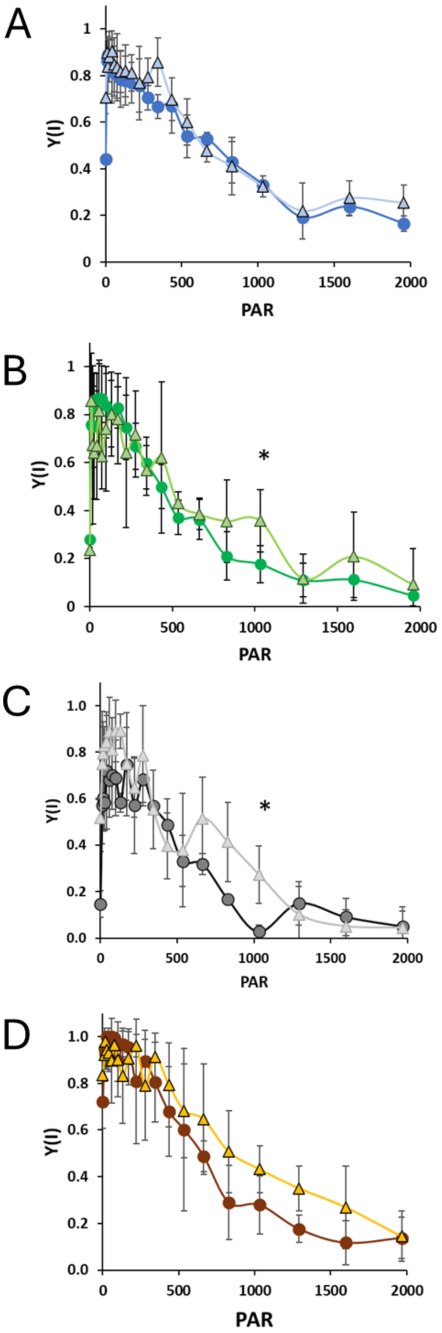

Table S1. Cell macromolecular composition.

The table lists the cellular protein content and macromolecular pool ratios showing the relative abundance of proteins, carbohydrates and lipids obtained from the deconvolution of FTIR spectra as described in Giordano et al., 2001 in *T. suecica*, *D. salina*, *D. salina* 3xNaCl and *P. tricornutum* control (CTR) and S-limited (S-lim) samples. The protein content and macromolecular pool ratios in the CTR and S-lim samples were compared and asterisks indicate when the values are statistically different (n≥3, t test, *, p<0.05; **, p<0.01; ***, p<0.001).

		CTR	S-lim	
T. suecica	Proteins (pg/cell)	50.2 ± 11.2	62.1 ± 10.3	**
	Lipids/Proteins (a.u.)	0.21 ± 0.02	0.24 ± 0.06	
	Lipids/Carbohydrates (a.u.)	0.040 ± 0.018	0.059 ± 0.008	**
	Carbohydrates/Proteins (a.u.)	6.2 ± 2.6	4.0 ± 1.1	*
D. salina	Proteins (pg/cell)	32.9 ± 4.9	38.9 ± 5.4	*
	Lipids/Proteins (a.u.)	0.11 ± 0.03	0.11 ± 0.04	
	Lipids/Carbohydrates (a.u.)	0.014 ± 0.004	0.010 ± 0.003	
	Carbohydrates/Proteins (a.u.)	8.3 ± 0.6	11.7 ± 3.2	**
***D. salina* 3xNaCl**	Proteins (pg/cell)	44.8 ± 4.2	54.9 ± 2.7	*
	Lipids/Proteins (a.u.)	0.07 ± 0.01	0.11 ± 0.01	***
	Lipids/Carbohydrates (a.u.)	0.013 ± 0.002	0.016 ± 0.004	*
	Carbohydrates/Proteins (a.u.)	5.6 ± 0.9	7.0 ± 1.2	*
P. tricornutum	Proteins (pg/cell)	7.7 ± 0.4	18.4 ± 1.5	***
	Lipids/Proteins (a.u.)	0.13 ± 0.02	0.15 ± 0.02	
	Lipids/Carbohydrates (a.u.)	0.109 ± 0.018	0.089 ± 0.023	
	Carbohydrates/Proteins (a.u.)	1.2 ± 0.1	1.7 ± 0.4	*
	Si/Proteins (a.u.)	0.37 ± 0.01	0.42 ± 0.04	*

Table S2. Fv/Fm and Pm values.

The table lists the values for maximum PSII quantum yield (Fv/Fm) and maximum P700 absorption signal (Pm) of *T. suecica*, *D. salina*, *D. salina* 3xNaCl and *P. tricornutum* samples grown in control (CTR) or S-limited (S-lim) conditions. Data are shown as mean ± standard deviation of at least 3 independent replicates. Asterisks indicate a significant difference between the S-lim sample and the respective CTR sample (t test, *, p<0.05; **, p<0.01; ***, p<0.001). Noteworthy, during the analyses of Chl fluorescence and P700+ absorption signal, CTR and S-lim samples were analysed based on an equal cell concentration. Therefore, the reduced Pm observed may be due to the different amount of Chl present in cells grown in CTR and S-lim conditions, as detailed in the text.

	**Fv/Fm**		**Pm**	
	**CTR**	**S-lim**	**CTR**	**S-lim**
T. suecica	0.68 ± 0.01	0.70 ± 0.03	0.085 ± 0.010	0.062 ± 0.004 *
D. salina	0.61 ± 0.02	0.63 ± 0.02	0.072 ± 0.020	0.033 ± 0.008 **
***D. salina* 3xNaCl**	0.69 ± 0.01	0.61 ± 0.02 ***	0.068 ± 0.012	0.030 ± 0.008 **
P. tricornutum	0.66 ± 0.02	0.61 ± 0.04 **	0.043 ± 0.010	0.041 ± 0.002

Table S3. Estimation of C fixation in control and S-limited cultures.

The table reports the estimation of C fixation in *T. suecica*, *D. salina*, *D. salina* 3xNaCl and *P. tricornutum* samples grown in control (CTR) or S-limited (S-lim) conditions. We calculated the pg C/ml of culture at the start of batch culture (t=0, $2.5*10^5$ cells/ml in the green microalgae, $5*10^5$ cells/ml in *P. tricornutum*) and at the sampling day in the mid-late exponential phase, assuming the composition of cells in the two time points is the same, as cultures in the exponential growth phase (i.e., in the same condition of the samples analysed to characterize cell composition), were used as starting material for experimental flasks. The pg C/ml at t=0 and at sampling day (t=3-6 days according to the species/culture conditions) were used to calculate the fold change of pg C/ml reported in the table, used to estimate the overall C fixation of the culture. pg C/ml/day was also calculated taking into account the different growth times of each sample. pg C/ml/day thus estimates the average C fixation per day of each species/culture condition during the exponential phase of growth. Data are shown as mean ± standard deviation of at least 3 independent replicates. Asterisks indicate when S-lim values differ from the respective CTR (t test, *, $p<0.05$; **, $p<0.01$; ***, $p<0.001$).

		CTR	S-lim
T. suecica	pgC ml^{-1}	7.2 ± 1.7	6.5 ± 0.9
	pgC ml^{-1} Day^{-1}	2.4 ± 0.6	2.2 ± 0.3
D. salina	pgC ml^{-1}	8.3 ± 2.3	7.2 ± 1.4
	pgC ml^{-1} Day^{-1}	2.8 ± 0.8	1.2 ± 0.2 ***
***D. salina* 3xNaCl**	pgC ml^{-1}	7.1 ± 0.5	4.8 ± 0.6 **
	pgC ml^{-1} Day^{-1}	2.4 ± 0.2	0.8 ± 0.1 ***
P. tricornutum	pgC ml^{-1}	11.1 ± 1.7	6.4 ± 1.4 *
	pgC ml^{-1} Day^{-1}	3.7 ± 0.6	2.1 ± 0.5 *
